# Comparative Genomic Analysis Revealed Distinct Molecular Components and Organization of CO_2_-Concentrating Mechanism in Thermophilic Cyanobacteria

**DOI:** 10.3389/fmicb.2022.876272

**Published:** 2022-05-06

**Authors:** Jie Tang, Huizhen Zhou, Dan Yao, Sadaf Riaz, Dawei You, Anna Klepacz-Smółka, Maurycy Daroch

**Affiliations:** ^1^School of Food and Bioengineering, Chengdu University, Chengdu, China; ^2^School of Environment and Energy, Peking University Shenzhen Graduate School, Shenzhen, China; ^3^Department of Bioprocess Engineering, Faculty of Process and Environmental Engineering, Łódź University of Technology, Łódź, Poland

**Keywords:** thermophilic cyanobacteria, inorganic carbon uptake, CO_2_-concentrating mechanisms (CCMs), carboxysomes, photosynthesis, Rubisco

## Abstract

Cyanobacteria evolved an inorganic carbon-concentrating mechanism (CCM) to perform effective oxygenic photosynthesis and prevent photorespiratory carbon losses. This process facilitates the acclimation of cyanobacteria to various habitats, particularly in CO_2_-limited environments. To date, there is limited information on the CCM of thermophilic cyanobacteria whose habitats limit the solubility of inorganic carbon. Here, genome-based approaches were used to identify the molecular components of CCM in 17 well-described thermophilic cyanobacteria. These cyanobacteria were from the genus *Leptodesmis*, *Leptolyngbya*, *Leptothermofonsia*, *Thermoleptolyngbya*, *Thermostichus*, and *Thermosynechococcus*. All the strains belong to β-cyanobacteria based on their β-carboxysome shell proteins with 1B form of Rubisco. The diversity in the C_i_ uptake systems and carboxysome composition of these thermophiles were analyzed based on their genomic information. For C_i_ uptake systems, two CO_2_ uptake systems (NDH-1_3_ and NDH-1_4_) and BicA for HCO_3_^–^ transport were present in all the thermophilic cyanobacteria, while most strains did not have the Na^+^/HCO_3_^–^ Sbt symporter and HCO_3_^–^ transporter BCT1 were absent in four strains. As for carboxysome, the β-carboxysomal shell protein, ccmK2, was absent only in *Thermoleptolyngbya* strains, whereas ccmK3/K4 were absent in all *Thermostichus* and *Thermosynechococcus* strains. Besides, all *Thermostichus* and *Thermosynechococcus* strains lacked carboxysomal β-CA, ccaA, the carbonic anhydrase activity of which may be replaced by ccmM proteins as indicated by comparative domain analysis. The genomic distribution of CCM-related genes was different among the thermophiles, suggesting probably distinct expression regulation. Overall, the comparative genomic analysis revealed distinct molecular components and organization of CCM in thermophilic cyanobacteria. These findings provided insights into the CCM components of thermophilic cyanobacteria and fundamental knowledge for further research regarding photosynthetic improvement and biomass yield of thermophilic cyanobacteria with biotechnological potentials.

## Introduction

Thermophilic cyanobacteria are photoautotrophic prokaryotes with a cosmopolitan distribution in diverse thermal environments (Tang et al., 2018a,b; Alcorta et al., 2020). With the increase of studies on these unique microorganisms and their niches, their importance as primary photosynthetic producers of the geothermal ecosystems becomes apparent. The thermophilic cyanobacteria account for a large part of the thermal ecosystem biomass and productivity. From an applicative perspective, thermophilic cyanobacteria can be explored for high value-added products relevant to agricultural, pharmaceutical, nutraceutical, and industrial applications (Patel et al., 2019). Particularly, the potential contribution of thermophilic cyanobacteria to mitigating carbon dioxide becomes attractive to researchers in the context of global warming and greenhouse gas emissions. Thermophilic cyanobacteria may facilitate the collection of CO_2_ right from locations where it was generated, e.g., fossil fuels power plants, thanks to their higher thermostability of photosynthetic apparatus and resistance to photosynthetic inhibitors such as NO_x_ and SO_x_ found in high levels in the flue gases (Liang et al., 2019; Chen and Wang, 2020).

The CO_2_-concentrating mechanism (CCM) is a vital biological process for CO_2_ utilization by cyanobacteria under conditions of CO_2_ limitation. Cyanobacterial CCM enables adequate photosynthetic CO_2_ fixation by elevating the CO_2_ level near the active site of Rubisco, thus providing adaptation to various CO_2_-limited niches (Clement et al., 2016; Raven et al., 2017). For most thermophilic cyanobacteria, the aquatic environments where they live suffer from low availability of inorganic carbon (Ci), primarily due to external factors, e.g., temperature, pH and gas exchange (Durall and Lindblad, 2015).

**FIGURE 1:**
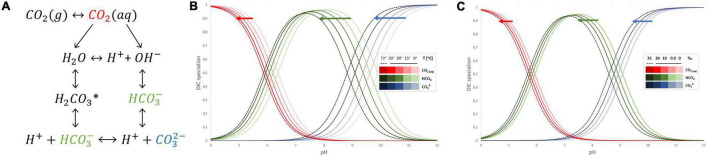
Interconversions of inorganic carbon species in aqueous solution **(A)**. The effect of temperature on inorganic carbon species distribution across different pH in aqueous solution **(B)**. The effect of salinity in ‰ on inorganic carbon species distribution across different pH in aqueous solution **(C)**.

The dissolution of gaseous CO_2_ in water starts from CO_2_ (g) transferring to CO_2_ (aq), i.e., CO_2_ mass transfer from gas to liquid phase (Figure 1A). Further, the small fraction of dissolved CO_2_ reacts in the liquid phase *via* two parallel paths depending on pH and subsequent availability of OH^–^. One leads to H^+^ and HCO_3_^–^ formation with the unstable intermediate product – carbonic acid H_2_CO_3_*, while the second with hydroxide ions also forms bicarbonate ions which further can dissociate into H^+^ and CO_3_^–^. When the pH of the aqueous solution is lower than 8, CO_2_ hydration follows the first path, and due to the very low value of the dissociation constant of the bicarbonate, the formation of carbonate ions can be neglected (Kierzkowska-Pawlak et al., 2022). The direct data on CO_2_ dissociation and different species of inorganic carbon present in hot springs have not been much discussed in the literature yet. However, the known equations of dissociation constants of CO_2_ in water and saline solutions are sufficient approximations to understand what inorganic carbon species are available across different pH ranges of those hot springs. The most commonly used equations for dissociation constants of CO_2_ in water (both fresh and saline) are applicable up to 40–50°C (Mook, 2000; Woosley, 2021). Based on those works, Bjerrum plot (Figure 1) of the carbonate system can be further extrapolated to 72°C and adjusted for salinity (Mook, 2000; Solomon, 2001; Tremaine et al., 2004). The solubility of CO_2_ in water is a function of temperature and do lower extend salinity (Figure 1), the higher the temperature, the lower the carbon dioxide solubility, elevating the temperature from 30 to 60°C results in 40% lower availability of CO_2_. Further decrease of that solubility occurs due to increased salinity or presence of other dissolved elements ubiquitous in hot springs. Meanwhile the distribution of carbon species across different pH is also affected by the temperature and to lesser extend salinity (Figure 1). Whilst bicarbonate (HCO_3_^–^) remains a dominant dissolved inorganic specie between pH 6 and 10; its ratio with other carbon species is temperature sensitive. The equilibrium between carbonic acid, bicarbonate and carbonate species is acid-shifted with an increase of temperature. Therefore, to survive in their unique aquatic habitat, the thermophilic cyanobacteria utilize CCM to ensure that the Rubisco with low affinity for CO_2_ is surrounded by high CO_2_ levels and regularly function regardless of thermal stress.

Prior studies on the effect of temperature on Rubisco, including that of thermophilic origin revealed that the maximal of rates of carboxylation reaction are typically higher than optimal growth temperatures of an organism (Galmés et al., 2015, 2016). This discrepancy in is partially due to the decreased affinity of Rubisco and lower specificity of carboxylation versus oxidation reaction at higher temperatures (S_c/o_) (Gubernator et al., 2008; Galmés et al., 2015, 2016). All in all thermophilic cyanobacteria must operate carbon concentration mechanisms to mitigate both challenges associated with lower carbon availability resulting from its poor solubility, and lower specificity of Rubisco toward CO_2_ at elevated temperatures.

In general, the cyanobacterial CCM comprises two primary components: C_i_ uptake systems and carboxysomes. Cyanobacteria have been reported to have up to five different systems to actively acquire and transport C_i_ into the cells, namely two for uptake of CO_2_ and three for transport of HCO_3_^–^ (Pronina et al., 2017). The two CO_2_ uptake systems that convert cytosolic CO_2_ into HCO_3_^–^ rely on plastoquinone oxidoreductase NADPH dehydrogenase respiratory complexes (Price, 2011). The complexes include NDH-1_3_, a low-CO_2_ inducible high-affinity CO_2_ uptake system encoded by *ndhD3*, *ndhF3* and *cupA* (*chpY*) genes, and NDH-1_4_, a constitutive low-affinity CO_2_ uptake system encoded by *ndhD4*, *ndhF4*, and *cupB* (*chpX*) genes (Kaplan, 2017). The transport of HCO_3_^–^ takes place at the plasma membrane and is performed by three transporters, including *bicA* (a *sulP*-type sodium-dependent HCO_3_^–^ transporter), *sbtA* (a sodium-dependent HCO_3_^–^ symporter), and *BCT1* (an ATP-binding cassette ABC-type HCO_3_^–^ transporter) (Raven et al., 2017). Inside the cyanobacterial cell, the HCO_3_^–^ is further transported into the carboxysomes that comprise protein shells and two encapsulated enzymes, Rubisco and carbonic anhydrase (CA) (Kerfeld and Melnicki, 2016). CA catalyzes the conversion of HCO_3_^–^ into CO_2_, which is a substrate for Rubisco. Rubisco consisting in cyanobacteria of eight small (*rbcS*) and eight large (*rbcL*) subunits, catalyzes CO_2_ fixation reaction to generate 3-phosphoglycerate for subsequent rearrangement through the Calvin-Benson-Bassham cycle (Hill et al., 2020). There are two types of shell proteins encoded by *cso* operon and *ccmKLMNO* operon, termed α-carboxysomes and β-carboxysomes, respectively. Based on this criterion, the cyanobacterial species carrying form 1A of Rubisco within α-carboxysomes are classified as α-cyanobacteria, while the species containing form 1B of Rubisco within β-carboxysomes as β-cyanobacteria (Melnicki et al., 2021). The *ccmP* was another β-carboxysome component, forming a bilayered shell protein (Cai et al., 2013). In addition, various carboxysomal CAs have been reported, including β-CA (*ccaA*) and γ-CA (*ccmM*) in β-cyanobacteria and β-CA (*csoSCA*) in α-cyanobacteria (MacCready et al., 2020). Two types of non-carboxysomal CAs were also reported in β-cyanobacteria, namely *ecaB* (β-CA) and *ecaA* (α-CA) (Sun et al., 2019).

Although CCM of freshwater, marine and alkaliphilic cyanobacteria have been reported (Burnap et al., 2015; Kupriyanova and Samylina, 2015; Klanchui et al., 2017), no comprehensive investigation on CCM of thermophilic cyanobacteria have been performed. Moreover, previous studies mainly focused on cyanobacterial CCM related to environments with distinct C_i_ content, salinity and especially pH that strongly influenced the CCM due to the equilibrium of C_i_ species (Mangan et al., 2016). Therefore, it is indispensable to survey the CCM of thermophilic strains in relation to their habitats that limit the solubility of inorganic carbon (Kamennaya et al., 2012). In recent years, next-generation sequencing (NGS) has been extensively employed to investigate the cyanobacterial genomes in thermal environments (Walter et al., 2017; Alcorta et al., 2020; Chen et al., 2021). This offers an opportunity to explore the thermophilic cyanobacterial CCM and the relationship with their niches at the genomic level. Furthermore, the studies on the molecular components of cyanobacterial CCM in relation to their specific habitats may improve photosynthetic CO_2_ fixation and biomass production in cyanobacteria (Noreña-Caro and Benton, 2018) and crop plants (Hennacy and Jonikas, 2020) through genetic engineering.

In the present study, genome sequences of 17 thermophilic cyanobacterial strains were used to investigate the molecular basis of CCM by computational identification. The relationship between CCM components and adaptations of these thermophilic cyanobacteria were further discussed. The insights into the CCM components lay a solid foundation for future research regarding photosynthetic improvement and biomass yield of thermophilic cyanobacteria with biotechnological potentials.

## Materials and Methods

### Genome Collection of Thermophilic Cyanobacteria

According to the genomic resources of the NCBI at the time of this study (2021/05/20), cyanobacteria with available genomes and closely related to thermophilic or hot-spring strains were first retrieved as a preliminary dataset. To ensure the accurate collection of thermophilic cyanobacteria, these strains were further filtered by conducting literature searches, contacting culture collections, leading authors of the manuscripts and submissions to retain thermophilic cyanobacteria with definite information about their thermophilic characteristics. Then, quality control for all genomes was performed based on the following criteria to reduce data redundancy and biased genome representation of cyanobacteria. First, the whole-genome average nucleotide identity (ANI) for each pair of genomes was calculated using the ANI calculator with default settings^1^. Genomes with an ANI value greater than 99.9% were considered as redundant genomes, and then only one of these genomes was randomly kept for the analysis. Second, the quality of the genomes was evaluated using CheckM (Parks et al., 2015) to ensure a more reliable genome dataset with near completeness (≥95%) and low contamination (<5%). These analyses finally generated a dataset comprising 16 thermophilic cyanobacteria. The genome, protein sequences and genomic annotations of the thermophilic cyanobacteria studied were downloaded from the database of NCBI. The genomes with no or incomplete annotations were annotated using the RAST annotation system (Aziz et al., 2008), provided in Supplementary Table 1.

Information regarding the 16 thermophilic strains was summarized in Table 1. Briefly, the 16 thermophiles were affiliated to four families of order Pseudanabaenales and Synechococcales, including Leptolyngbyaceae: *Leptodesmis sichuanensis* A121 (Tang et al., 2022a), *Leptolyngbya* sp. JSC-1 (Brown et al., 2010), *Leptothermofonsia sichuanensis* E412 (Tang et al., 2022b); Oculatellaceae: *Thermoleptolyngbya* sp. O-77 (Yoon et al., 2017), *T. sichuanensis* A183 (Tang et al., 2021); Synechococcaceae: *Synechococcus* sp. 60AY4M2, 63AY4M2, 65AY6A5, and 65AY6Li (Olsen et al., 2015), *Thermostichus* sp. JA-2-3B and JA-3-3Ab (Bhaya et al., 2007); Thermosynechococcaceae: *Thermosynechococcus* sp. CL-1 (Cheng et al., 2020) and TA-1 (Leu et al., 2013), *T. vestitus* BP-1 (Nakamura et al., 2002) and E542 (Liang et al., 2019), *T. vulcanus* NIES-2134 (Liang et al., 2018).

**TABLE 1:** Ecological information and genome characteristics of thermophilic cyanobacteria studied.

Species	Isolation source	Niche temperature (°C)	Niche pH	Genome completeness (%)	Genome contamination (%)	Accession number	References
*Leptodesmis sichuanensis* A121	Hot spring, Erdaoqiao, Sichuan, China	40.8°C	6.32	99.53	0.94	GCA_021379005	Tang et al., 2022a
*Leptolyngbya* sp. JSC-1	La Duke Hot Springs, Montana, United States	60°C	6.85	99.53	1.30	GCA_000733415	Brown et al., 2010
*Leptothermofonsia sichuanensis* E412	Hot spring, Lotus Lake, Sichuan, China	42.7°C	8.61	99.29	0.00	GCA_019891175	Tang et al., 2022b
*Thermoleptolyngbya sichuanensis* A183	Hot spring, Erdaoqiao, Sichuan, China	40.8°C	6.32	98.94	0.94	GCA_013177315	Tang et al., 2021
*Thermoleptolyngbya* sp. O-77	Hot spring, Kumamoto, Japan	35–60°C	NA	98.70	1.53	GCA_001548395	Yoon et al., 2017
*Thermostichus* sp. 60AY4M2	Mushroom Spring, Yellowstone National Park, United States	60°C	alkaline	100.00	0.88	GCA_002760375	Olsen et al., 2015
*Thermostichus* sp. 63AY4M2	Mushroom Spring, Yellowstone National Park, United States	63°C	alkaline	100.00	0.00	GCA_002760475	Olsen et al., 2015
*Thermostichus* sp. 65AY6A5	Mushroom Spring, Yellowstone National Park, United States	65°C	alkaline	100.00	0.88	GCA_002760415	Olsen et al., 2015
*Thermostichus* sp. 65AY6Li	Mushroom Spring, Yellowstone National Park, United States	65°C	alkaline	99.61	1.32	GCA_002760345	Olsen et al., 2015
*Thermostichus* sp. JA-2-3Ba	Octopus Spring, Yellowstone National Park, United States	58–65°C	8.5	100.00	0.00	GCA_000013225	Bhaya et al., 2007
*Thermostichus* sp. JA-3-3Ab	Octopus Spring, Yellowstone National Park, United States	58–65°C	8.5	100.00	1.32	GCA_000013205	Bhaya et al., 2007
*Thermosynechococcus lividus* PCC 6715	Hot spring, Yellowstone National Park, United States	NA	NA	98.86	0.12	GCA_002754935	This study
*Thermosynechococcus* sp. CL-1	Chin-Lun hot spring, Taiwan, China	62°C	9.3	100.00	0.12	GCA_008386235	Cheng et al., 2020
*Thermosynechococcus* sp. TA-1	Taian hot springs, Taiwan, China	50°C	7–9	100.00	0.12	GCA_017086385	Leu et al., 2013
*Thermosynechococcus vestitus* BP-1	Hot spring, Beppu, Japan	52°C	7.5	99.76	0.12	GCA_000011345	Nakamura et al., 2002
*Thermosynechococcus vestitus* E542	Hot spring, Lotus lake, Sichuan, China	67.2°C	7.95	100.00	0.12	GCA_003555505	Liang et al., 2019
*Thermosynechococcus vulcanus* NIES-2134	Yunomine Hot Spring, Wakayama, Japan	50–57°C	NA	99.76	0.12	GCA_003990665	Liang et al., 2018

### Genome Sequencing and *de novo* Assembly

Additional to the 16 downloaded genomes, we sequenced the genome of another thermophilic cyanobacterium, purchased from Pasteur Culture Collection of Cyanobacteria as *Synechococcus lividus* PCC 6715. Whole-genome sequencing was carried out using integrated sequencing strategies, including PacBio and Illumina HiSeq Technology. The PacBio SMRT sequencing of PCC 6715 yielded 96,587 adapter-trimmed reads (subreads) with an average read length of approximately 11 kbp, corresponding to 400-fold coverage. *De novo* assembly was performed using the hierarchical genome assembly process (HGAP) method implemented in SMRT analysis v2.3.0 (Chin et al., 2013), generating two contigs. The Illumina sequencing of PCC 6715 generated 5,566,668 filtered paired-end reads (clean data), providing approximately 520-fold coverage of the genome. The clean data of short reads generated by Illumina were assembled into contigs using SOAPdenovo v2.04 (Luo et al., 2012) with default parameters. Based on the contigs from SOAPdenovo assembler, the contigs derived from the HGAP method were comparatively examined to determine their continuity and concatenated into one closed circular chromosome. The genome obtained was mapped with Illumina reads using BWA v0.7.17 (Li and Durbin, 2009) and then Pilon v1.23 (Walker et al., 2014) to correct any assembly and sequence errors. The genome of PCC 6715 was annotated as described above and deposited in the NCBI under the accession number CP018092.

### Identification of Orthologous Proteins

Amino acid sequences of 28 proteins involved in CCM of *Synechocystis* sp. PCC 6803 were downloaded from the CyanoBase^2^ as a reference protein set. Based on the bidirectional best hit (BBH) criterion (Brilli et al., 2010), orthologous proteins involved in CCM of the studied species were identified using BLASTP with the following thresholds: *E*-value cut-off of 1E-6, ≥30% identity and 70% coverage. The identified proteins were homologous to these proteins in *Synechocystis* PCC 6803, namely *bicA1* (*sll0834*), *bicA2* (*slr0096*), *ccaA* (*slr1347*), *ccmK1* (*sll1029*), *ccmK2* (*sll1028*), *ccmK3* (*slr1838*), *ccmK4* (*slr1839*), *ccmL* (*sll1030*), *ccmM* (*sll1031*), *ccmN* (*sll1032*), *ccmO* (*slr0436*), *ccmP* (*slr0169*), *cmpA* (*slr0040*), *cmpB* (*slr0041*), *cmpC* (*slr0043*), *cmpD* (*slr0044*), *cupA* (*sll1734*), *cupB* (*slr1302*), *ecaB* (*slr0051*), *ndhD3* (*sll1733*), *ndhD4* (*sll0027*), *ndhF3* (*sll1732*), *ndhF4* (*sll0026*), *rbcL* (*slr0009*), *rbcS* (*slr0012*), *rbcX* (*slr0011*), *sbtA* (*slr1512*), and *SbtB* (*slr1513*). The homologs of *ecaA* protein were searched using the experimentally confirmed protein *ecaA* (*all2929*) of *Anabaena* PCC 7120 (Soltes-Rak et al., 1997) as a query. The italic number in bracket mentioned above refers to the gene ID in CyanoBase (see text footnote 2). The accession numbers of orthologous proteins identified in each genome of the thermophilic cyanobacteria studied was summarized in Supplementary Table 2.

### Phylogeny

Amino acid sequences of Rubisco large subunit (*rbcL*) were collected for the studied species and reference cyanobacteria. The phylogram of *rbcL* was used to infer the protein function and classification among the strains. Protein sequences of genes *ccmK*, -*L*, -*M*, -*N*, -*O*, and -*P* encoding carboxysome shell proteins, *cmpA*, -*B*, -*C*, and -*D* encoding the HCO_3_^–^ transporter BCT1 and *nrtA*, -*B*, -*C*, and -*D* of the nitrite/nitrate assimilation were also collected for phylogenetic reconstruction. All the protein sequences used were retrieved from the public databases, CyanoBase (see text footnote 2) or the NCBI^3^. All the phylogenetic analyses were performed using the following pipeline: multiple sequence alignments by Muscle complemented in Mega7 (Kumar et al., 2016) and Maximum-Likelihood (ML) inference of phylogenies by PhyML v3.3 (Guindon et al., 2010). Parameter settings in PhyML and bootstrap analysis of phylogenies were followed as described (Tang et al., 2019).

## Results and Discussion

### Thermophilic Cyanobacteria and Classification of Their Carbon-Concentrating Mechanism

The 17 thermophilic cyanobacteria collected in this study all originated from hot springs (Table 1). Although the niche temperatures showed significant variation, these thermophilic cyanobacteria were defined based on their ability to grow above 50°C. Two types of morphology were characterized for these thermophilic cyanobacteria, comprising unicellular *Synechococcus*, *Thermostichus* and *Thermosynechococcus*, and filamentous *Leptodesmis*, *Leptolyngbya*, *Leptothermofonsia*, and *Thermoleptolyngbya*. In addition, strains JA-2-3Ba and JA-3-3Ab, previously known as *Synechococcus* strains, have been reclassified into a newly delineated taxon, genus *Thermostichus* (Komárek et al., 2020). According to the suggested values for species (ANI > 96%, AAI ≥ 95%) and genus (ANI < 83%, AAI ≤ 70%) delimitation (Jain and Rodriguez, 2018), the result of genome-wide ANI and AAI indicated that strain JA-2-3Ba and JA-3-3Ab were different species (ANI = 85.6%, AAI = 87.8%) within *Thermostichus*, while strain JA-3-3Ab, plus the other four strains from Yellowstone National Park (Table 1), belong to the same species (ANI/AAI > 98%) also within *Thermostichus*. However, the case of *S. lividus* PCC 6715 was contradictory. Between *S. lividus* PCC 6715 and the five *Thermosynechococcus* strains, the values of ANI and ANI were ∼ 77 and ∼ 81% (Supplementary Table 3), respectively. To resolve the dilemma, a third approach was employed to determine the percentages of conserved proteins (POCP) between genomes. The POCP values (Supplementary Table 3) between *S. lividus* PCC 6715 and the *Thermosynechococcus* strains ranged from 83.4 to 84.0%, all far beyond the threshold (50%) for the definition of a prokaryotic genus (Qin et al., 2014). Taken together, strain PCC 6715 might be a member of *Thermosynechococcus*, and was proposed to be *Thermosynechococcus lividus*. However, this conclusion requires validation with comprehensive investigation beyond the genomic data.

**FIGURE 2:**
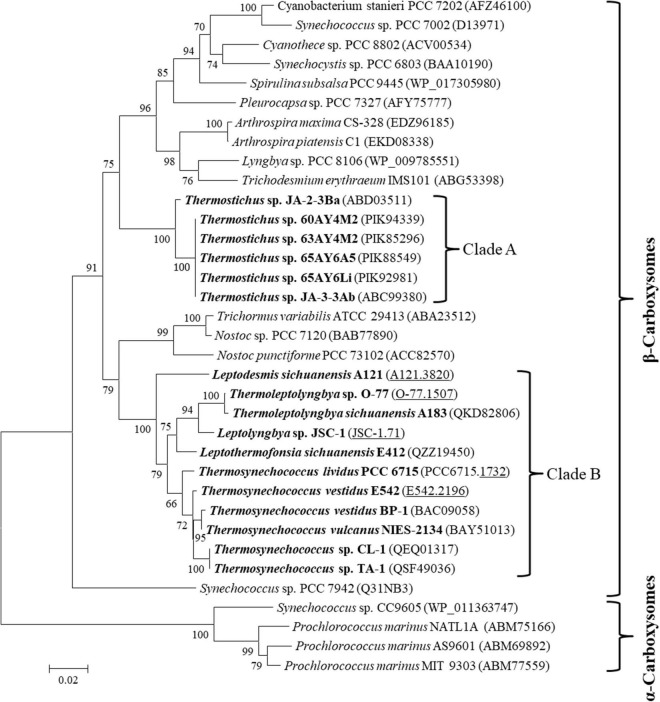
Phylogenetic inference of Rubisco large subunit protein sequences. The thermophilic cyanobacteria investigated in this study are indicated in bold. The accession numbers underscored refers to the gene IDs in Supplementary Table 1. Only bootstrap values > 50% are indicated at nodes. Scale bar = 1% substitutions per site.

The carboxysome type of the 17 thermophilic cyanobacteria was investigated based on the phylogenetic analysis of a frequently used molecular marker, *rbcL* (Klanchui et al., 2017). The ML phylogram of *rbcL* categorized the 35 cyanobacteria into two categories (Figure 2), α-cyanobacteria and β-cyanobacteria, according to their Rubisco forms. There was only one exception that *Synechococcus* PCC 7942 was solely placed in a separate branch. The 17 thermophilic cyanobacteria surveyed were all grouped into the β-cyanobacteria category, indicating the presence of Rubisco 1B form in these thermophiles. However, considerable genetic diversity of *rbcL* amino acid sequences was also shown among these thermophilic cyanobacteria, distributing the strains into two clades (Figure 2). Clade A included only *Thermostichus* strains, also known as early-branching or early-divergent *Synechococcus* (Blank and Sánchez-Baracaldo, 2010), whereas clade B contained thermophiles from a different genus. In addition, the clustering of strains did not completely comply with morphology, e.g., filamentous strains clustered with unicellular strains (clade B). Intriguingly, the 17 thermophilic strains did not group with any non-thermophilic strains, suggesting *rbcL* specificity of thermophiles. Although phylogenetic analysis of *rbcL* sequences can be used for the classification of cyanobacterial types, the evolutionary relationship based on habitats or morphology cannot be elucidated from the present *rbcL* phylogram. This is consistent with the conclusions in previous studies that phylogenetic inference of molecular markers may be improper for the elaboration of evolutionary relationship with cyanobacterial habitat and environments (Komárek, 2016; Klanchui et al., 2017).

### Genes Encoding C_i_ Uptake Systems in Thermophilic Cyanobacteria

Five different transport systems (Table 2) have been identified in the 17 thermophilic cyanobacteria. The two CO_2_ uptake systems, NDH-1_3_ and NDH-1_4_ complex, were present in all the surveyed thermophilic cyanobacteria. Both CO_2_ uptake systems in these thermophilic cyanobacteria were consistent with the previous reports that both CO_2_ transporters were present in β-cyanobacteria living in freshwater, brackish or eutrophic lakes (Sandrini et al., 2014; Durall and Lindblad, 2015). In contrast, oceanic α-cyanobacteria (e.g., *Prochlorococcus* species) and marine β-cyanobacteria (e.g., *Trichodesmium* species) contained only one or even lacked them entirely (Price et al., 2007; Pronina et al., 2017). These results strongly indicated that the presence of NDH-1_3_ and NDH-1_4_ might be relevant to the environments where these cyanobacteria live. Thermophilic cyanobacteria, similar to freshwater and estuarine strains, contained essential components of CO_2_ uptake and bicarbonate transport genes. Both constitutive systems NDH-1_4_ complex and *bic*A were ubiquitous in all known thermophilic strains. The inducible NDH-1_3_ complex is also universal suggesting that these strains are prepared for occasional carbon dioxide shortages. Unfortunately, it was not verified based on the current data that the niche temperature directly affected the existence of the two CO_2_ uptake systems in light of their near-ubiquity in non-thermophilic cyanobacteria. The protein sequences of five genes encoding the two systems showed different identities with the sequences of non-thermophilic reference cyanobacteria (*Synechocystis* PCC 6803), ranging from 50.4 to 64.7% (Table 2), but a high degree of homology (≥70% identity) was noticed only among *cupA* genes. In addition, intragenus and intergenic variations of protein sequences were revealed by the different identities (Table 2). Moreover, the distinct property of the two complexes, a low-CO_2_ inducible high-affinity CO_2_ uptake system (NDH-1_3_ complex) and a constitutive low-affinity CO_2_ uptake system (NDH-1_4_ complex), may confer the thermophilic cyanobacteria with more alternative strategies to survive in environments with significant CO_2_ fluctuation, particularly in hot springs.

**TABLE 2:** Availability of genes encoding Ci uptake systems of thermophilic cyanobacteria studied.

Strain	Ci uptake systems													
	CO_2_ uptake							HCO_3_^–^ transport						
	NDH-1_4_ complex			NDH-1_3_ complex			BicA		Sbt regulator		BCT1			
	*ndhD4*	*ndhF4*	*cupB*	*ndhD3*	*ndhF3*	*cupA*	*bicA1*	*bicA2*	*sbtA*	*sbtB*	*cmpA*	*cmpB*	*cmpC*	*cmpD*
*Leptodesmis* A121	√ (60.9)	√ (59.7)	√ (62.7)	√ (61.5)	√ (57.2)	√ (71.8)	√ (65.8)	x	x	x	√ (69.2)	√ (70.9)	√ (73.6)	√ (67.2)
*Leptolyngbya* JSC-1	√ (62.6)	√ (56.9)	√ (62.9)	√ (59.6)	√ (59.0)	√ (81.7)	√ (57.3)	x	√ (75.9)	√ (79.1)	√ (63.9)	√ (65.2)	√ (70.8)	√ (72.2)
*Leptothermofonsia* E412	√ (63.8)	√ (59.3)	√ (64.7)	√ (62.8)	√ (60.8)	√ (84.2)	√ (64.8)	√ (59.2)	x	x	√ (63.0)	√ (64.5)	√ (70.8)	√ (73.6)
*Thermoleptolyngbya* A183	√ (60.3)	√ (55.6)	√ (62.3)	√ (58.8)	√ (57.8)	√ (85.7)	√ (65.5)	√ (62.3)	√ (70.7)	√ (62.5)	x	x	x	x
*Thermoleptolyngbya* O-77	√ (59.0)	√ (56.1)	√ (61.3)	√ (57.7)	√ (57.7)	√ (84.7)	√ (65.5)	x	√ (71.9)	√ (63.4)	√ (68.7)	√ (64.8)	√ (78.2)	√ (75.8)
*Thermostichus* 60AY4M2	√ (57.6)	√ (54.5)	√ (56.8)	√ (58.6)	√ (53.8)	√ (75.8)	√ (60.2)	x	x	x	√ (65.8)	√ (65.9)	√ (72.2)	√ (66.8)
*Thermostichus* 63AY4M2	√ (57.4)	√ (54.5)	√ (56.8)	√ (58.6)	√ (53.9)	√ (75.8)	√ (60.2)	x	x	x	√ (65.8)	√ (66.2)	√ (72.1)	√ (66.8)
*Thermostichus* 65AY6A5	√ (57.4)	√ (54.5)	√ (56.8)	√ (58.6)	√ (53.8)	√ (75.8)	√ (59.8)	x	x	x	√ (65.8)	√ (66.2)	√ (72.2)	√ (66.8)
*Thermostichus* 65AY6Li	√ (57.4)	√ (54.5)	√ (56.8)	√ (58.4)	√ (53.9)	√ (75.3)	√ (60.0)	x	x	x	√ (65.4)	√ (66.2)	√ (72.2)	√ (67.1)
*Thermostichus* JA-2-3Ba	√ (57.6)	√ (53.6)	√ (59.6)	√ (58.1)	√ (53.4)	√ (76.0)	√ (59.8)	x	x	x	√ (68.2)	√ (67.0)	√ (71.7)	√ (63.3)
*Thermostichus* JA-3-3Ab	√ (57.4)	√ (54.5)	√ (56.8)	√ (58.4)	√ (53.9)	√ (75.3)	√ (60.2)	x	x	x	√ (67.5)	√ (66.2)	√ (71.8)	√ (65.0)
*Thermosynechococcus* PCC 6715	√ (56.8)	√ (55.5)	√ (59.7)	√ (59.2)	√ (50.4)	√ (70.7)	x	√ (57.1)	√ (81.6)	√ (80.9)	x	x	x	x
*Thermosynechococcus* CL-1	√ (59.3)	√ (56.7)	√ (61.9)	√ (61.5)	√ (58.1)	√ (70.6)	x	√ (57.2)	√ (74.1)	√ (80.9)	√ (66.5)	√ (59.5)	√ (73.1)	√ (71.2)
*Thermosynechococcus* TA-1	√ (60.2)	√ (56.8)	√ (62.6)	√ (61.1)	√ (58.0)	√ (70.8)	x	√ (56.8)	x	√ (80.2)	x	x	x	x
*Thermosynechococcus* BP-1	√ (60.3)	√ (56.8)	√ (61.4)	√ (61.4)	√ (57.7)	√ (70.7)	x	√ (56.1)	x	x	√ (67.7)	√ (61.0)	√ (73.3)	√ (66.8)
*Thermosynechococcus* E542	√ (59.9)	√ (57.4)	√ (62.0)	√ (62.9)	√ (59.0)	√ (70.4)	x	√ (56.4)	√ (72.7)	√ (81.8)	x	x	x	x
*Thermosynechococcus* NIES-2134	√ (59.9)	√ (56.8)	√ (61.4)	√ (61.6)	√ (58.3)	√ (70.7)	x	√ (53.2)	x	x	√ (67.5)	√ (68.1)	√ (73.0)	√ (68.1)

*√ and x refers to the presence and absence of the gene, respectively. The number in brackets indicates the identity (%) between the identified protein and the corresponding sequence of Synechocystis PCC 6803.*

**FIGURE 3:**
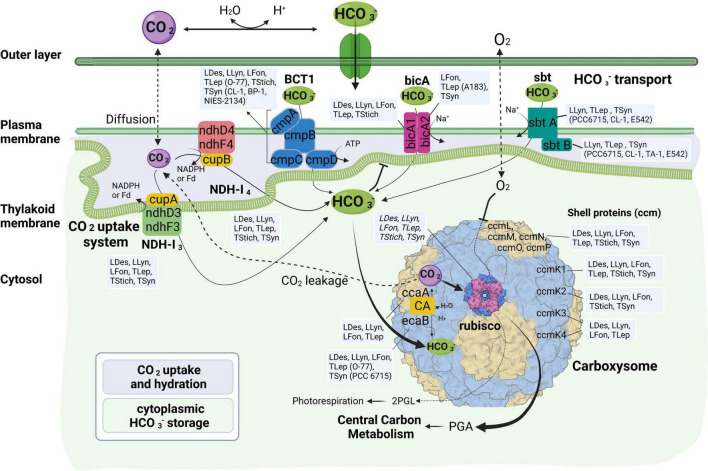
Molecular components of CCM characteristic for various strains of thermophilic cyanobacteria divided according to their function. LDes – *Leptodesmis*, LLyn – *Leptolyngbya*, LFon – *Leptothermofonsia*, TLep – *Thermoleptolyngbya*, TStich – *Thermostichus*, TSyn – *Thermosynechococcus*; 2PGL – 2-phosphoglycolate; PGA – phosphoglyceric acid. Figure created with BioRender.com.

More variations among the 17 thermophilic cyanobacteria were noticed in HCO_3_^–^ transport systems than in CO_2_ uptake systems (Table 2 and Figure 3). Association of the abundance of these mechanisms with their respective environmental niches is inconclusive. Similar repertoires of CO_2_ and bicarbonate uptake genes were observed in *L. sichuanensis* A121 with the lowest habitat temperature and the most thermophilic strains of genus *Thermostichus* (Table 2 and Figure 3). When it comes to the pH of the environmental niches, no evident niches preference of genes was observed. Genetic repertoire of strains isolated from acidic and alkaline niches appeared to be unrelated to the niche pH. For *bicA* transporter, only *Leptothermofonsia* E412 and *Thermoleptolyngbya* A183 possessed both *bicA1* and *bicA2*, while the other strains contained *bicA1* or *bicA2* (Table 2). The amino acid sequence identities of *bicA* genes were around 60% between the thermophilic cyanobacteria and *Synechocystis* PCC 6803. The presence of *bicA1* and/or *bicA2* was consistent within each genus except for *Thermoleptolyngbya* (strain A183 and O-77). Sequence analysis suggested that a putative protein-coding gene of O-77 (O-77.5066, Supplementary Table 1) showed very low amino acid coverage (46.9%) to *bicA2* of A183, but the aligned region was almost identical to each other (Supplementary Figure 1), indicating this short CDS might be a partial region of *bicA2*. Such discrepancy in CDS length of *bicA2* within *Thermoleptolyngbya* requires future studies to elucidate the possible causes, e.g., mutations, gene lost, or sequencing errors. For *sbt* regulator, both *sbtA* and *sbtB* were found only in six thermophilic cyanobacteria (Table 2). Furthermore, considerable variations existed within the genus *Thermosynechococcus*. Strain E542, PCC 6715 and CL-1 possessed both *sbtA* and *sbtB*. TA-1 had only *sbtB*, while the other two *Thermosynechococcus* strains contained none. This result implied that the *sbt* genes of these *Thermosynechococcus* strains might be acquired by horizontal gene transfer or vertically inherited but lost during the evolutionary process. In addition, it appeared that the thermophilic cyanobacteria typically have *bicA* rather than *sbt* as revealed by the dominant presence (Table 2). This could be ascribed to the distinct traits of the two transporters and the habitat of these cyanobacteria. First, *bicA* shows low affinity and high flux rate to HCO_3_^–^ and the genes encoding *bicA* were found to be primarily constitutively expressed (Price et al., 2004). Second, *sbt* was highly inducible under carbon-limited conditions and had a relatively high affinity to HCO_3_^–^ (Price, 2011). The *sbt* may therefore become accessory when cyanobacteria live in alkaline ecosystems typically rich in HCO_3_^–^, which was in accordance with most cases in this study (Table 1), e.g., *Thermostichus* strains. Meanwhile, it was evident that thermophilic cyanobacteria with both transporters might be advantageous to acclimatize to the highly dynamic shift of exogenous HCO_3_^–^ by selective utilization of the two HCO_3_^–^ transport systems.

**FIGURE 4:**
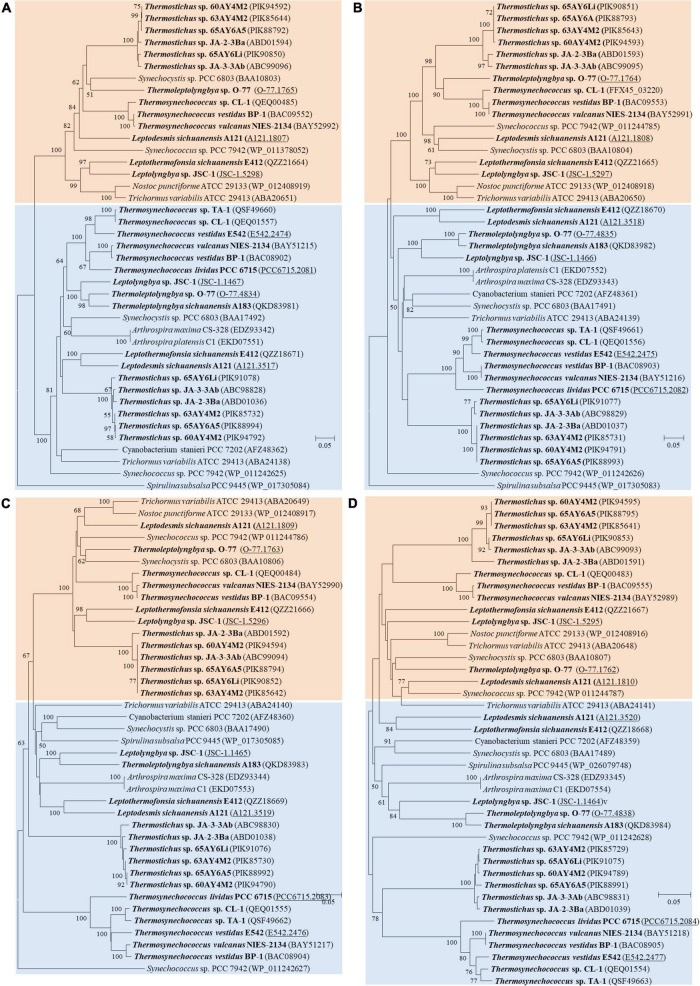
Phylogenetic inference of protein sequences of *cmp* and *nrt* genes. **(A)** *cmpA* and *nrtA*; **(B)** *cmpB* and *nrtB*; **(C)** *cmpC* and *nrtC*; **(D)** *cmpD* and *nrtD*. The thermophilic cyanobacteria investigated in this study are indicated in bold. The accession numbers underscored refers to the gene IDs in Supplementary Table 1. Only bootstrap values > 50% are indicated at nodes.

As for the third HCO_3_^–^ transporter, BCT1 encoded by *cmpA*, -*B*, -*C*, and -*D* and with high affinity for HCO_3_^–^ was induced under low levels of C_i_ and enhanced by high light conditions (Omata et al., 2002). The *cmp* genes were detected in all thermophilic cyanobacteria except for *Thermoleptolyngbya* A183 and *Thermosynechococcus* E542, PCC 6715 and TA-1 (Table 2). Intriguingly, strains without *cmp* genes all possessed *sbt*, which might to some extent compensate the function of *cmp* genes for adaptation to thermal environments limited in inorganic carbon (Kamennaya et al., 2012). The identified *cmp* genes showed amino acid sequence identity of 59.5–75.8% with the reference proteins (Table 2). In addition, it was reported that *nrtA*, -*B*, -*C*, and -*D* (nitrate/nitrite transport system) shared high sequence similarity with *cmpA*, -*B*, -*C*, and -*D* (Omata, 1995). Therefore, we also identified the *nrt* genes in the 17 thermophilic cyanobacteria, and phylogenetic analysis based on amino acid sequences of *cmp* and *nrt* genes was performed to verify the annotations with the help of experimentally confirmed *cmp* proteins of *Synechococcus* PCC 7942 (Omata et al., 1999). As shown in Figure 4, the *cmp* proteins of thermophilic cyanobacteria clustered with that of *Synechococcus* PCC 7942 and other reference strains, while the *nrt* proteins grouped into clades or clusters separated from *cmp* proteins. Furthermore, sequence conservation was low, as suggested by long branches among the clusters formed in the phylogram (Figure 4). Surprisingly, the genome of *Thermoleptolyngbya* O-77 was the only genome without the complete set of *nrtA*, -*B*, -*C*, and -*D*, *nrtC* of which was not found by BLASTP. However, sequence analysis (Supplementary Figure 2) showed that two putative genes both shared high similarity of protein sequence with *nrtC* of *Thermoleptolyngbya* A183, suggesting unknown interruption within CDS of *nrtC* in O-77 genome. This might be ascribed to mutations, partial gene lost, or sequencing errors. Intriguingly, the discrepancy of the presence of *cmp* genes within genus *Thermoleptolyngbya* and *Thermosynechococcus* again suggested that these genes might be acquired by horizontal gene transfer or vertically inherited but lost during the evolutionary process.

In summary, the C_i_ uptake systems could assign the 17 thermophilic cyanobacteria into three genotypes: (I) strains comprising NDH-1_3_, NDH-1_4_, *bicA*, *sbt*, and BCT1; (II) NDH-1_3_, NDH-1_4_, *bicA*, and *sbt*; and (III) NDH-1_3_, NDH-1_4_, *bicA*, and BCT1. This result suggested that the same CO_2_ uptake systems and different HCO_3_^–^ transport systems were shared by these thermophilic cyanobacteria. Different genotypes were primarily attributed to the presence/absence of *sbt* and BCT1 (Table 2). Only three strains, namely *Leptolyngbya* JSC-1, *Thermoleptolyngbya* O-77, and *Thermosynechococcus* CL-1, contained both types of high-affinity HCO_3_^–^ transporters, indicating that these strains may have a higher capacity for HCO_3_^–^ uptake under various growth environments. Definitely, owning one of *sbt* and BCT1 appeared to be sufficient for thermophilic strains to cope with extremely low inorganic carbon and ions in thermal aquatic environments. Future experimental studies should be carried out to elucidate the function of these *in silico* identified proteins in thermophilic cyanobacteria.

### Genes Encoding Carboxysomes in Thermophilic Cyanobacteria

**FIGURE 5:**
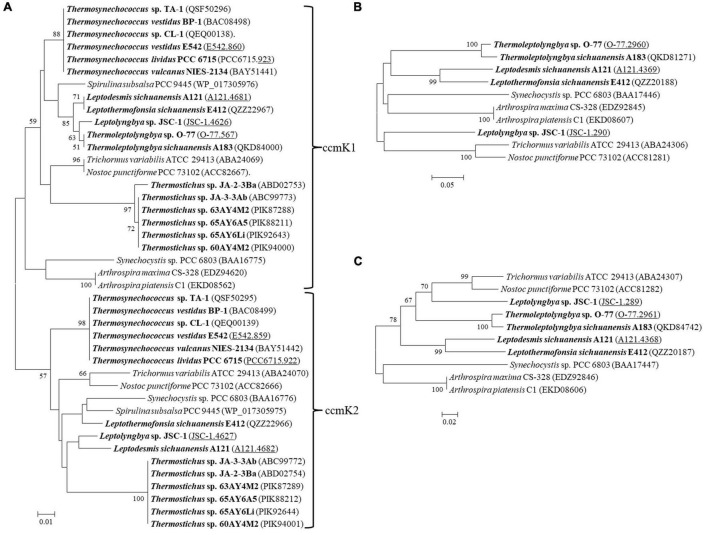
Phylogenetic inference of protein sequences of *ccmK1/2* **(A)**, *ccmK3* **(B)** and *ccmK4* **(C)**. The thermophilic cyanobacteria investigated in this study are indicated in bold. The accession numbers underscored refers to the gene IDs in Supplementary Table 1. Only bootstrap values > 50% are indicated at nodes.

Carboxysomes in cyanobacteria are bacterial microcompartments (BMCs) that provide an environment for enhancing the catalytic capabilities of the CO_2_ fixing enzyme, Rubisco (Cai et al., 2009). A typical β-cyanobacterial genome usually contains *ccmK1*-*4* (MacCready et al., 2020). However, only three out of 17 thermophilic cyanobacteria studied showed such diversity of the shell proteins, while *Thermoleptolyngbya* strains lacked *ccmk2*, and *Thermostichus* and *Thermosynechococcus* strains contained none of *ccmk3* and *ccmK4* (Table 3). Previous studies showed that *ccmK1* and *ccmK2* were the main structural proteins of the carboxysome shell (Kerfeld et al., 2005; Rae et al., 2013). Phylogenetic analysis and identity calculation suggested particularly high sequence conservation between the two homologs of *ccmK1/2*, as revealed by low amino acid substitution rates and short branches (Figure 5A) and high identity (Table 3). In contrast, phylogenetic branches of the *ccmK3* and *ccmK4* proteins were much longer, and residues were less conserved (Figures 5B,C and Table 3). Although deletion of the *ccmK2* gene in *Synechococcus* PCC 7942 that lacked *ccmK1* caused a carboxysome-less phenotype (Rae et al., 2012), this experiment did not verify whether normal phenotype can be achieved with the presence of sole *ccmK1* homolog. Besides, the main distinguishing feature between *ccmK1* and *ccmK2* was the ∼10 amino acid long C-terminal extension of *ccmK1* (Kerfeld et al., 2005), which has been proven to show limited structural relevance for shell assembly (Cai et al., 2016). Instead, it might be just the genomic proximity of the two *ccmK* genes, as indicated by its high degree of conservation (15/17 genomes, Table 3). Future studies are required to carefully investigate the specificity regarding the structure and function of *ccmk1* in the *Thermoleptolyngbya* strains. Regarding *ccmK3* and *ccmK4*, the widespread absence of the two genes was not surprising. The previous studies showed they are similar molecular components (Sommer et al., 2017) and suggested functional redundancy that play only a minor structural role (Rae et al., 2012). On the other hand, a recent study proposed a functional scenario in which minor *ccmK3/K4* incorporation in shells would introduce sufficient local disorder to allow shell remodeling (Garcia-Alles et al., 2019). Another study indicated that *ccmK3*-*ccmK4* heterohexamers potentially expand the range of permeability properties of metabolite channels in carboxysome shells (Sommer et al., 2019). Taken together, these findings suggested that *ccmK3/K4* present in *Leptodesmis* A121, *Leptolyngbya* JSC-1, *Leptothermofonsia* E412, and *Thermoleptolyngbya* A183 and O-77 may be utilized to adjust the properties of carboxysome for rapid adaptation to environmental changes that happen in thermal regions. In addition to *ccmK*, other genes encoding carboxysome shell proteins, *ccmL*, -*M*, -*N*, -*O*, and -*P* were also found to be present in all 17 thermophilic cyanobacteria (Table 3). Only *ccmN* exhibited weak homologs (<40%). All the surveyed thermophilic cyanobacteria showed a high degree of homology (67.6–76.1%) with the *ccmP* of *Synechocystis* PCC 6803, and similarly identity range of 65.8–75.5% to the *ccmP* of *Synechococcus* PCC 7942, an experimentally confirmed protein (Cai et al., 2013). Further phylogenetic analysis suggested extensive genetic diversity in these genes as indicated by the assignments of these thermophilic cyanobacteria into different clusters or clades (Supplementary Figure 3).

**TABLE 3:** Availability of genes encoding carboxysomes of thermophilic cyanobacteria studied.

Strain	Carboxysome													
	Shell proteins									Encapsulated enzymes				
	β-Carboxysomal shell proteins									Rubisco			β-CA	
	*ccmK1*	*ccmK2*	*ccmK3*	*ccmK4*	*ccmL*	*ccmM*	*ccmN*	*ccmO*	*ccmP*	*rbcL*	*rbcS*	*rbcX*	*ccaA*	*ecaB*
*Leptodesmis* A121	√ (85.2)	√ (91.3)	√ (56.9)	√ (64.8)	√ (56.4)	√ (49.5)	√ (35.0)	√ (54.2)	√ (72.0)	√ (85.1)	√ (63.9)	√ (41.7)	√ (49.7)	√ (39.5)
*Leptolyngbya* JSC-1	√ (84.3)	√ (91.3)	√ (62.9)	√ (61.5)	√ (67.6)	√ (47.1)	√ (37.9)	√ (58.6)	√ (73.7)	√ (84.3)	√ (68.1)	√ (42.2)	√ (48.1)	√ (41.0)
*Leptothermofonsia* E412	√ (85.2)	√ (94.2)	√ (63.8)	√ (61.8)	√ (57.8)	√ (48.9)	√ (38.2)	√ (54.0)	√ (72.8)	√ (83.9)	√ (63.9)	√ (42.9)	√ (48.6)	√ (38.1)
*Thermoleptolyngbya* A183	√ (85.2)	x	√ (63.1)	√ (63.1)	√ (66.0)	√ (44.9)	√ (37.8)	√ (55.6)	√ (75.6)	√ (83.7)	√ (67.8)	√ (39.4)	√ (39.5)	x
*Thermoleptolyngbya* O-77	√ (85.2)	x	√ (63.6)	√ (63.1)	√ (66.0)	√ (41.3)	√ (37.4)	√ (54.9)	√ (76.1)	√ (83.5)	√ (67.8)	√ (38.9)	√ (39.3)	√ (42.8)
*Thermostichus* 60AY4M2	√ (82.6)	√ (88.3)	x	x	√ (60.4)	√ (49.9)	√ (37.8)	√ (52.4)	√ (67.6)	√ (86.3)	√ (65.0)	√ (44.7)	x	x
*Thermostichus* 63AY4M2	√ (82.6)	√ (88.3)	x	x	√ (60.4)	√ (49.5)	√ (37.8)	√ (52.1)	√ (67.6)	√ (86.3)	√ (65.0)	√ (44.7)	x	x
*Thermostichus* 65AY6A5	√ (82.6)	√ (88.3)	x	x	√ (60.4)	√ (49.7)	√ (38.1)	√ (52.4)	√ (67.6)	√ (86.3)	√ (65.0)	√ (44.7)	x	x
*Thermostichus* 65AY6Li	√ (82.6)	√ (88.3)	x	x	√ (60.4)	√ (49.6)	√ (37.8)	√ (52.1)	√ (67.6)	√ (86.3)	√ (65.0)	√ (44.7)	x	x
*Thermostichus* JA-2-3Ba	√ (81.7)	√ (88.3)	x	x	√ (63.7)	√ (50.3)	√ (37.7)	√ (53.9)	√ (69.0)	√ (87.2)	√ (64.2)	√ (43.9)	x	x
*Thermostichus* JA-3-3Ab	√ (82.6)	√ (88.3)	x	x	√ (60.4)	√ (49.6)	√ (37.6)	√ (52.1)	√ (67.6)	√ (86.3)	√ (65.0)	√ (44.7)	x	x
*Thermosynechococcus* PCC 6715	√ (86.1)	√ (90.3)	x	x	√ (53.0)	√ (42.7)	√ (37.5)	√ (54.6)	√ (67.3)	√ (83.9)	√ (62.7)	√ (44.7)	x	√ (42.8)
*Thermosynechococcus* CL-1	√ (86.1)	√ (90.3)	x	x	√ (54.1)	√ (46.0)	√ (38.8)	√ (54.3)	√ (72.8)	√ (84.9)	√ (63.1)	√ (46.0)	x	x
*Thermosynechococcus* TA-1	√ (86.1)	√ (90.3)	x	x	√ (54.1)	√ (46.0)	√ (38.7)	√ (54.9)	√ (72.8)	√ (84.9)	√ (61.9)	√ (45.3)	x	x
*Thermosynechococcus* BP-1	√ (86.1)	√ (90.3)	x	x	√ (56.3)	√ (45.8)	√ (35.7)	√ (55.0)	√ (72.3)	√ (84.1)	√ (63.3)	√ (46.3)	x	x
*Thermosynechococcus* E542	√ (86.1)	√ (90.3)	x	x	√ (54.1)	√ (45.7)	√ (36.4)	√ (54.4)	√ (72.8)	√ (84.7)	√ (63.9)	√ (45.3)	x	x
*Thermosynechococcus* NIES-2134	√ (86.1)	√ (90.3)	x	x	√ (54.1)	√ (45.8)	√ (35.7)	√ (53.9)	√ (72.3)	√ (84.3)	√ (63.3)	√ (46.3)	x	x

*√ and x refers to the presence and absence of the gene, respectively. The number in brackets indicates the identity (%) between the identified protein and the corresponding sequence of Synechocystis PCC 6803.*

**FIGURE 6:**
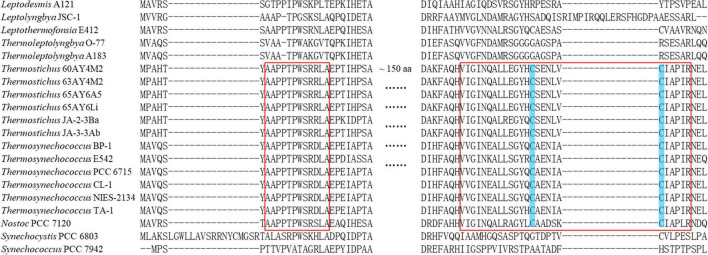
Partial alignments of *ccmM* amino acid sequences representing 17 thermophilic cyanobacteria, *Nostoc* PCC 7120, and *Synechocystis* PCC 6803 and *Synechococcus* PCC 7942 as outgroup. As suggested by Peña et al. (2010), red boxes refer to conserved regions of the N-terminal domain of *ccmM* necessary for CA activity, while shaded cysteine amino acids indicate essential residues participating in the disulfide bond in the C-termini of active *ccmM* protein.

The subunits of Rubisco, *rbcL* and *rbcS*, and Rubisco assembly chaperone, *rbcX*, were present in all the 17 thermophilic cyanobacteria (Table 3). The protein sequences of *rbcL* in thermophilic cyanobacteria were highly conserved to the reference protein (>83% identity), while that of *rbcS* was moderately conserved (∼ 65% identity). The protein sequences of *rbcX* were more variable, showing ∼ 45% identity with the reference protein. In regards to β-CA, carboxysomal *ccaA* and/or non-carboxysomal *ecaB* proteins with low sequence identity (38.1–49.7%) were detected only in six thermophilic strains (Table 3), respectively. Apart from the functions as shell protein, an additional function of *ccmM* (γ-CA) as CA activity was reported in cyanobacteria lacking *ccaA* (Peña et al., 2010; de Araujo et al., 2014). Thus, we compared γ-CA-like domain in N-terminal of *ccmM* in thermophilic cyanobacteria to the functional γ-CA in *Thermosynechococcus* BP-1 (Peña et al., 2010) and *Nostoc* PCC 7120 (de Araujo et al., 2014) as well as non-functional γ-CA in *Synechocystis* PCC 6803 (Cot et al., 2008) and *Synechococcus* PCC 7942 (So and Espie, 2005). As shown in Figure 6, strains from *Thermostichus* and *Thermosynechococcus* exhibited similar primary structure of amino acid residues in γ-CA-like domain of *ccmM* to that with CA activity in *Thermosynechococcus* BP-1 and *Nostoc* PCC 7120. The result suggested that these *ccmM* proteins may play a role in CA activity in light of the absence of a carboxysomal CA (*ccaA*) (Table 3) in these thermophilic cyanobacteria. Although strains from *Leptodesmis*, *Leptolyngbya*, *Leptothermofonsia*, and *Thermoleptolyngbya* had both *ccmM* and *ccaA*, it is likely that only *ccaA* function as CA activity as indicated by the primary structure analysis, which was consistent with the result found in haloalkaliphilic cyanobacteria *Microcoleus* sp. IPPAS B-353 (Kupriyanova et al., 2016). In addition, strains living in high temperatures above 50°C (Table 1) appeared to tend to lose *ccaA*. Meanwhile, high pH conditions were speculated to be an important factor in *ccaA* absence (Klanchui et al., 2017). However, the reasons were still unclear. Certainly, the co-evolution of *ccmM* and *ccaA* is crucial for carboxysome to regularly function in extreme environments regardless of high temperature or alkalinity. As for α-CA, non-carboxysomal *ecaA* was not found in all the 17 thermophilic cyanobacteria.

### Genomic Distribution Pattern of Carbon-Concentrating Mechanism-Related Genes in Thermophilic Cyanobacteria

**FIGURE 7:**
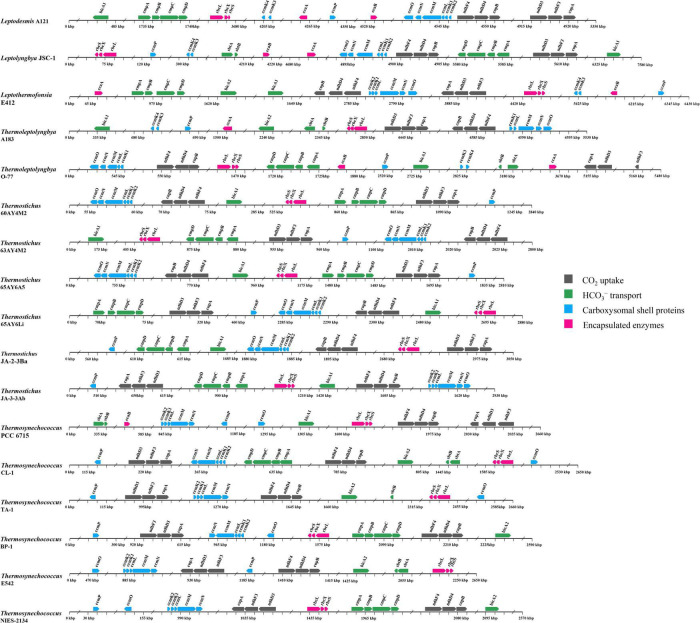
Genomic organization of CCM-related genes in the 17 thermophilic cyanobacteria studied. Solid arrow boxes refer to genes and the direction of transcription.

The genomic location of CCM-related genes in thermophilic cyanobacteria may provide insights into the function and evolution of these genes. In the present study, the genomic organization of CCM-related genes was illustrated in Figure 7. Genes encoding each NDH-1 complex were all clustered together, except for the *ndhD4* of *Thermosynechococcus* TA-1. This gene was remote from the other genes of NDH-1_4_ complex (Figure 7). In light of the coordination of *NDH* and *cup* proteins in CO_2_ uptake (Han et al., 2017), whether and how such organization in *Thermosynechococcus* TA-1 affects the co-regulation requires further experimental elucidation. In addition, the genes encoding *bicA1* and *bicA2* were positionally scattered in the genomes of *Leptothermofonsia* E412 and *Thermoleptolyngbya* A183, whereas genes encoding *cmpABCD* and *sbtA/B* were located together in genomes (Figure 7), respectively.

Focusing on the gene organization of the carboxysome, there appeared to be the main carboxysome locus (MCL) in the genomes of all the studied thermophilic cyanobacteria, comprising one to two *ccmK1/2* genes, always followed by *ccmL*, -*M*, -*N* and in the majority of genomes (11 out of 17), *ccmO* (Figure 7). The sequential arrangement of *ccmK2* and *ccmK1* in the MCL might facilitate protein complex assembly or help to balance the shell protein stoichiometry during translation of MCL genes (Cai et al., 2016). Moreover, previous studies suggested that *ccmK*, -*L*, -*M*, and -*N* were co-regulated in an MCL as an operon (Rae et al., 2013; Billis et al., 2014). In addition to the MCL, satellite loci were found in several genomes. Satellite locus encoding the *ccmP* protein was found in all the 17 thermophilic cyanobacteria. The satellite loci formed by *ccmK3* and *ccmK4* was present only in five genomes (Figure 7). The organization of *K1*/*K2* and *K3/K4* paralogs in separated operons might relate to structural segregation of the two groups (Sommer et al., 2019). Although *ccmK3* and *ccmK4* clustered in operon, it was reported that the stoichiometry of incorporation of *ccmK3* in associations with *ccmK4* was low in *Synechocystis* PCC6803 (Garcia-Alles et al., 2019). However, the expression of *ccmK3* and *ccmK4* from a satellite locus may increase the flexibility of carboxysome shell assembly and permeability (Sommer et al., 2019), and may provide differing metabolite selectivities (Sommer et al., 2017). Another satellite locus, *ccmO*, was positionally variable (Figure 7). In 11 genomes, *ccmO* was the terminal gene in the MCL. However, *ccmO* in six *Thermosynechococcus* genomes was located as a satellite locus, remote from other carboxysome genes, and this *ccmO* formed a distinct clade as revealed by the phylogenetic analysis (Supplementary Figure 3D). Previous transcriptome analysis demonstrated that the *ccmO* gene was co-regulated with MCL genes only when it was associated with the MCL (Sommer et al., 2017). Therefore, the dispersal of *ccmO* as well as other carboxysome genes across the genome may increase the plasticity of individual regulation of their expression, perhaps in response to changes in environmental conditions, and vice versa, diverse environmental conditions appear to promote relocation of these genes driven by evolutionary force (Cai et al., 2012; Shih et al., 2013).

Genes encoding *rbcL*, *rbcS*, and *rbcX* for Rubisco were localized together in all of the 17 thermophilic cyanobacteria, which was in line with the previous consensus that the three genes were clustered in an operon in β-cyanobacteria (Badger et al., 2002). The Rubisco gene cluster was all apart from the MCL in each genome, indicating that it, as satellite loci to MCL, might regulate its expression independently. This was supported by the results reported by Billis et al. (2014) that *ccmO*, *rbcL*, and *rbcS* were less strictly co-regulated with the MCL genes. As for the chaperone *rbcX*, sometimes it was non-essential for the assembly of an active Rubisco enzyme, e.g., in *Synechococcus* PCC 7942 (Emlyn-Jones et al., 2006). The role of *rbcX* in these thermophilic cyanobacteria requires future investigations. In regards to β-CAs, scattered distribution of *ccaA* and *ecaB* was present in the genomes.

### Functional and Comparative Analysis of Carbon-Concentrating Mechanism Components Among β-Cyanobacteria

The overall composition of the CCM system presented in Figure 3 contains all typical components found in other cyanobacteria, including the complete set of genes for carboxysome assembly. A comparison of these components among β-cyanobacteria was performed between thermophilic cyanobacteria and non-thermophilic cyanobacteria. For a precise comparison, we divided the non-thermophilic cyanobacteria into three groups: freshwater, marine, and alkaliphilic cyanobacteria. The overall compositions of CCM components in thermophilic cyanobacteria were more similar to the freshwater and alkaliphilic than the marine groups (Klanchui et al., 2017). The thermophilic, freshwater and alkaliphilic cyanobacteria possessed both CO_2_ uptake systems, NDH-1_3_ and NDH-1_4_, whereas most marine cyanobacteria lacked the NDH-1_3_. In light of the HCO_3_^–^ uptake, marine and some thermophilic/alkaliphilic cyanobacteria lacked the BCT1 transporter. Although freshwater cyanobacteria consistently possessed BCT1 transporter, some thermophilic cyanobacteria with freshwater origins lacked that. The relatively high diversity of bicarbonate transporters is a distinguishing factor for certain thermophilic strains probably as indicator of their specific location and not their thermophilic character. However, there is no apparent correlation between the pH of the isolation sources of these strains and the presence or absence of specific bicarbonate uptake systems (Figure 3 and Table 1). More thorough studies are required to characterize the functions of these transporters in the future. In addition, the freshwater cyanobacteria possessed the highest abundance of CAs, β-CA (*ccaA* and *ecaB*), α-CA (*ecaA*), and γ-CA (*ccmM*), while the thermophilic cyanobacteria with freshwater origins were likely to possess up to two CAs (Table 3 and Figure 3). Notably, 11 out of the 17 investigated thermophiles appeared to have only γ-CA (*ccmM*). On the other hand, the diversity of carboxysome shell proteins appears to be lower in thermophilic strains than many of their mesophilic counterparts, with multiple strains lacking at least one of the four *ccmK* genes.

## Conclusion

In the present study, we investigated the molecular components and distribution of CCM in 17 thermophilic cyanobacteria. The diversity in the C_i_ uptake systems and carboxysome of these thermophiles were observed. Particularly, the presence of HCO_3_^–^ transporters, *ccmK2/3/4* and CAs tremendously varied among these thermophiles. Several strains have more C_i_ uptake-related components, indicating the capabilities of these strains to acclimate and establish competitive growth by using different C_i_ uptake strategies in response to environmental fluctuation. The distinct genomic distribution of CCM-related genes among the thermophiles probably suggested various regulations of expression. Overall, the comparative genomic analysis revealed distinct molecular components and organization of CCM in thermophilic cyanobacteria. These findings provided insights into the CCM components of thermophilic cyanobacteria and fundamental knowledge for further research regarding photosynthetic improvement and biomass yield of thermophilic cyanobacteria with biotechnological potentials.

## Data Availability Statement

The datasets presented in this study can be found in online repositories. The names of the repository/repositories and accession number(s) can be found below: https://www.ncbi.nlm.nih.gov/, CP018092.

## Author Contributions

JT: conceptualization, methodology, validation, formal analysis, investigation, data curation, writing – original draft, writing, review, editing, visualization, supervision, project administration, and funding acquisition. HZ: formal analysis, investigation, data curation, and visualization. DYa: formal analysis, investigation, and data curation. SR: formal analysis, data curation, and visualization. DYo: formal analysis, visualization, writing, review, and editing. AK-S: formal analysis, investigation, and funding acquisition. MD: conceptualization, methodology, resources, data curation, writing – original draft, writing, review, editing, supervision, project administration, and funding acquisition. All authors contributed to the article and approved the submitted version.

## Conflict of Interest

The authors declare that the research was conducted in the absence of any commercial or financial relationships that could be construed as a potential conflict of interest.

## Publisher’s Note

All claims expressed in this article are solely those of the authors and do not necessarily represent those of their affiliated organizations, or those of the publisher, the editors and the reviewers. Any product that may be evaluated in this article, or claim that may be made by its manufacturer, is not guaranteed or endorsed by the publisher.

## Footnotes

1
http://enve-omics.ce.gatech.edu/ani/

2
http://genome.kazusa.or.jp/cyanobase

3
https://www.ncbi.nlm.nih.gov/
