# Supplementary material for: Comparative Genomic Analysis Revealed Distinct Molecular Components and Organization of CO2-Concentrating Mechanism in Thermophilic Cyanobacteria
Source: Front Microbiol. 2022 May 6;13:876272. doi: 10.3389/fmicb.2022.876272 (PMC9120777; doi:10.3389/fmicb.2022.876272)
Supplement: Supplementary file 1 [file Presentation_1.pdf]

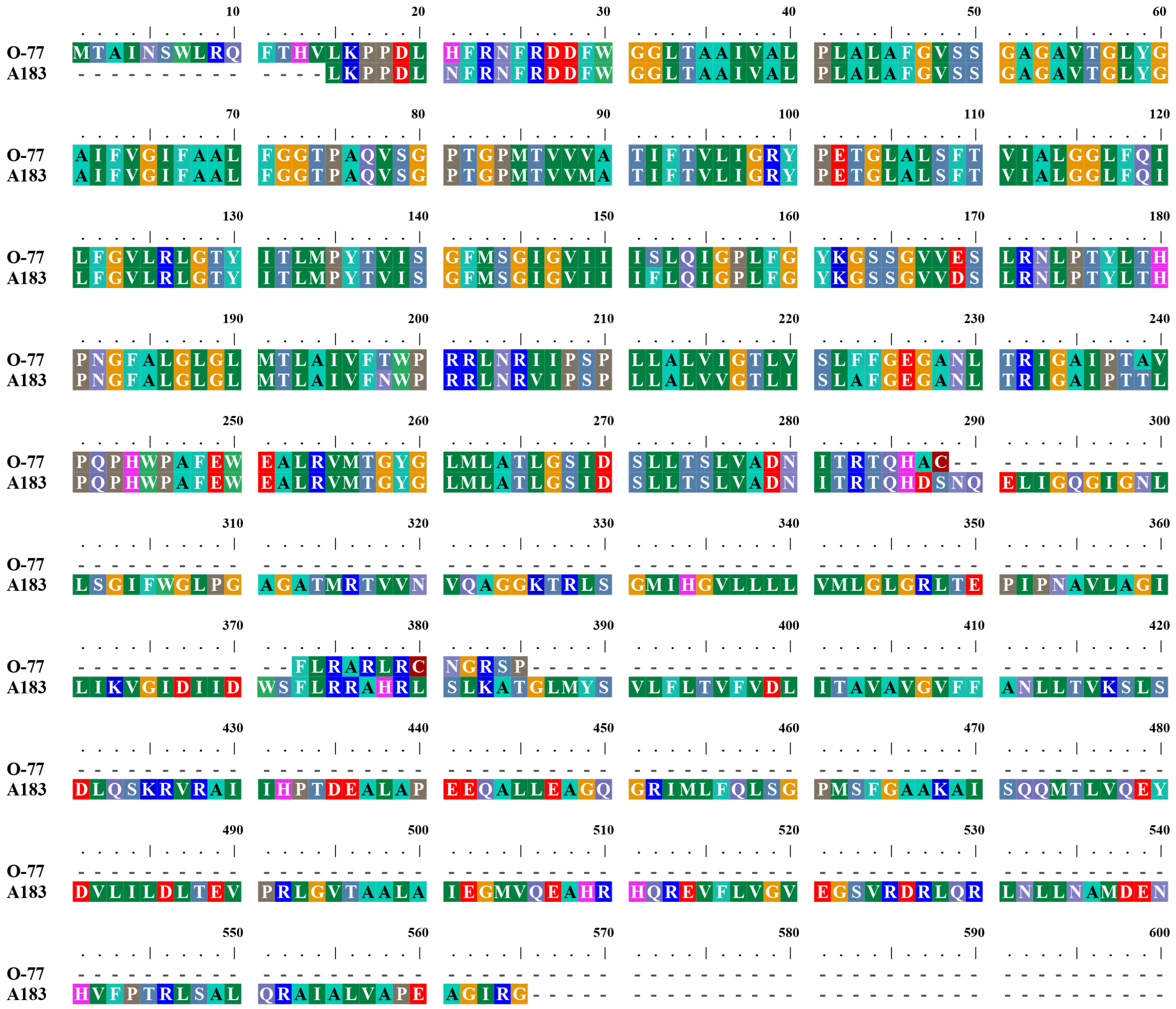

Supplementary Figure 1 Protein alignment of putative *bicA2* between two *Thermoleptolyngbya* strains. The gene id was O-77.5066 for O-77 (Supplementary Table 1) and QKD84875 for A183 (Supplementary Table 2), respectively.

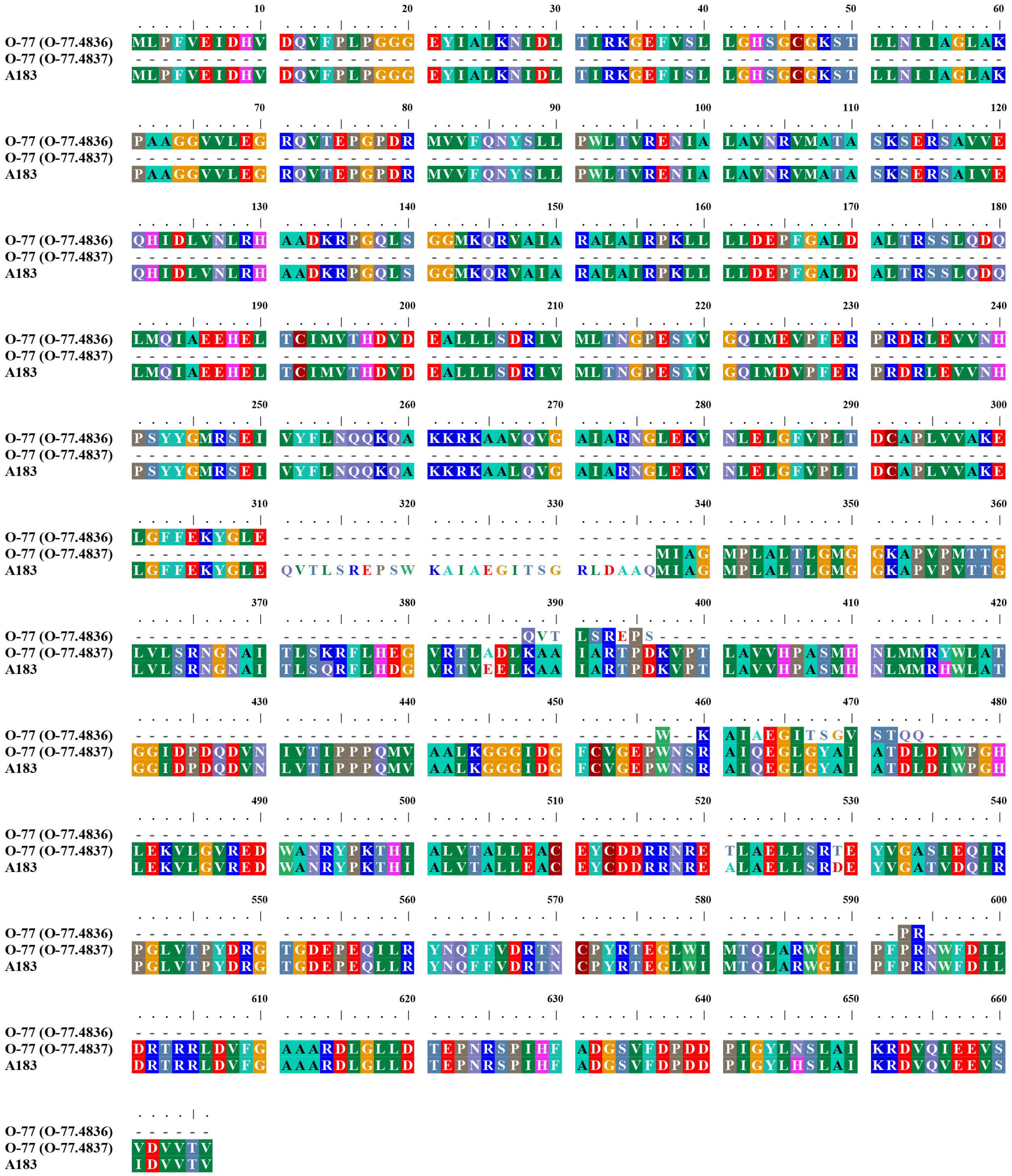

Supplementary Figure 2 Protein alignment of putative *nrtC* between two *Thermoleptolyngbya* strains. The gene id was O-77.4836 and O-77.4837 for O-77 (Supplementary Table 1) and QKD83983 for A183 (Supplementary Table 2), respectively.

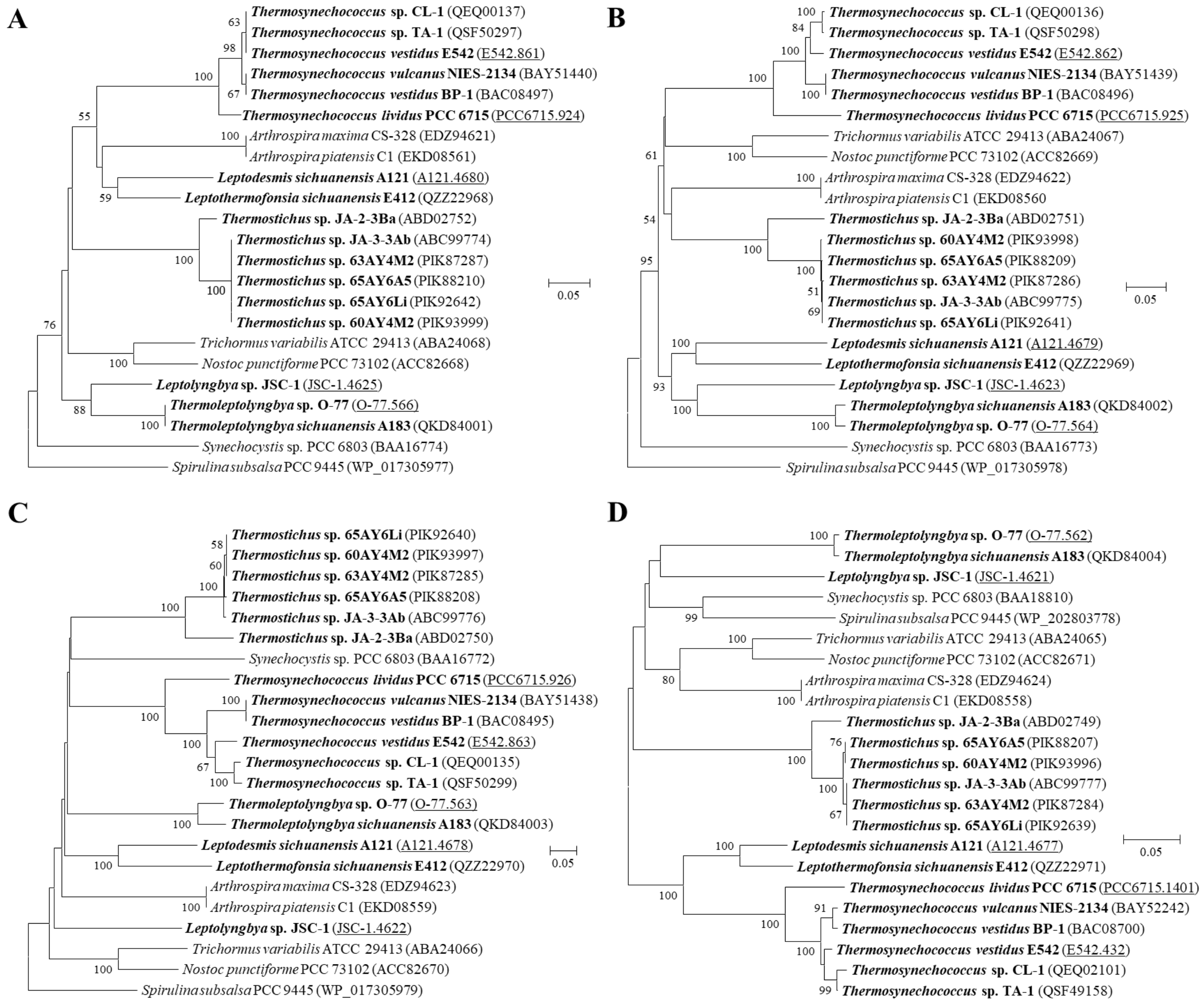

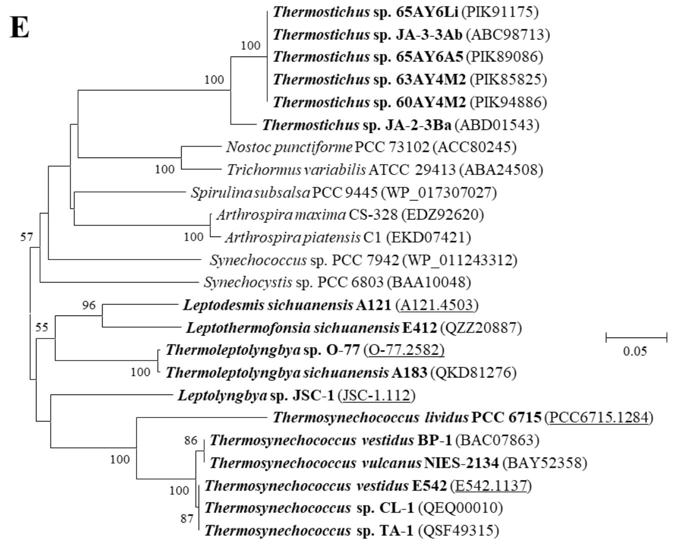

Supplementary Figure 3 Phylogenetic inference of protein sequences of *ccmL* **(A)**, *ccmM* **(B)**, *ccmN* **(C),** *ccmO* **(D)** and *ccmP* **(E)**. The thermophilic cyanobacteria investigated in this study are indicated in bold. The accession numbers underscored refers to the gene IDs in Supplementary Table 1. Only bootstrap values>50% are indicated at nodes.
